# Nerve Repair Using Decellularized Nerve Grafts in Rat Models. A Review of the Literature

**DOI:** 10.3389/fncel.2018.00427

**Published:** 2018-11-19

**Authors:** Arianna B. Lovati, Daniele D’Arrigo, Simonetta Odella, Pierluigi Tos, Stefano Geuna, Stefania Raimondo

**Affiliations:** ^1^Cell and Tissue Engineering Laboratory, IRCCS Istituto Ortopedico Galeazzi, Milan, Italy; ^2^UOC Hand Surgery and Reconstructive Microsurgery Unit, ASST G. Pini-CTO, Milan, Italy; ^3^Department of Clinical and Biological Sciences, San Luigi Gonzaga Hospital, University of Turin, Turin, Italy

**Keywords:** nerve injury, nerve regeneration, allograft, decellularized nerve graft, rat model

## Abstract

Peripheral nerve regeneration after severe traumatic nerve injury is a relevant clinical problem. Several different strategies have been investigated to solve the problem of bridging the nerve gap. Among these, the use of decellularized nerve grafts has been proposed as an alternative to auto/isografts, which represent the current gold standard in the treatment of severe nerve injury. This study reports the results of a systematic review of the literature published between January 2007 and October 2017. The aim was to quantitatively analyze the effectiveness of decellularized nerve grafts in rat experimental models. The review included 33 studies in which eight different decellularization protocols were described. The decellularized nerve grafts were reported to be immunologically safe and able to support both functional and morphological regeneration after nerve injury. Chemical protocols were found to be superior to physical protocols. However, further research is needed to optimize preparation protocols, including recellularization, improve their effectiveness, and substitute the current gold standard, especially in the repair of long nerve defects.

## Introduction

Traumatic injury of peripheral nerves is of considerable clinical importance due to its high incidence. The estimated incidence is upward of 300,000 cases per year in Europe (Kingham and Terenghi, 2006) and more than one million per year worldwide (Daly et al., 2012). Peripheral nerve lesions are five times more frequent than spinal cord lesions and results in decreased or complete loss of sensitivity and/or motor activity. The ability of peripheral nerves to regenerate has been recognized for more than a century; however, clinical and experimental evidence shows that regeneration is often unsatisfactory, especially following severe injury (Navarro et al., 2007; Sun et al., 2009; Pfister et al., 2011). For treating mild injury, direct suturing is the most common surgical technique. For nerve substance loss, nerve autograft (transplantation of an autologous nerve) is currently the gold standard (Battiston et al., 2009) despite its limitations (e.g., additional surgery, scarring, and donor-site morbidity, limited source of donor nerves) (Hoben et al., 2018). There is a strong need to find innovative therapies.

Recent research has focused on the use of biological and synthetic conduits. Progress in materials sciences, with the availability of new biomaterials and innovative manufacturing procedures (Siemionow et al., 2013) has led to the development of innovative artificial conduits. However, as the translation of synthetic nerve grafts from bench to bedside is still limited, biological tubulization remains the mainstay approach. Albert (1885) first used a nerve graft from a donor (allograft) to reconstruct a damaged nerve after resection of a sarcoma. Although devoid of the drawbacks of autografts, allografts can be rejected by the recipient body and therefore require systemic immunosuppressive therapy. Advances in tissue engineering in the last decade have led to the realization of decellularized nerve allografts (Hundepool et al., 2017; Philips et al., 2018). Unlike conventional allografts, they are cleaned of their antigenic component yet retain their 3D structure which serves as scaffolds for axonal growth. Decellularized nerve allografts could thus represent a potentially ideal alternative.

The aim of this review was to quantitatively analyze the relevant scientific literature for comparative assessment of the effectiveness of nerve allografts in relation to decellularized grafts.

## Methods

**FIGURE 1:**
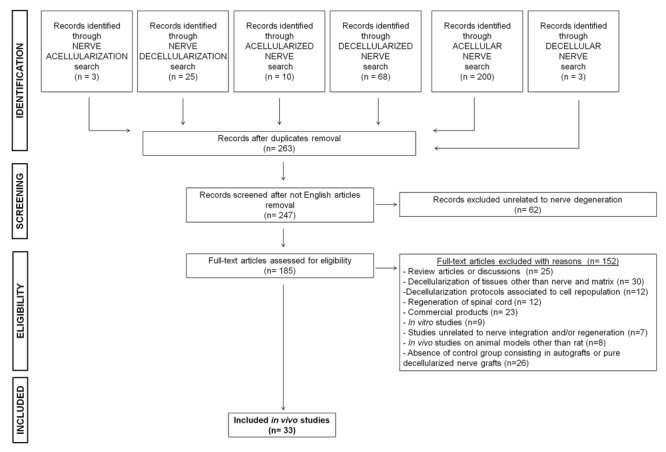
Research strategy. Flow chart of the selection process.

The PubMed database was searched for articles published in English between January 2007 and October 2017. The search terms were “decellularization, decellularized, decellular, acellularization, acellular, acellularized” combined with the keyword “nerve.” The present study was focused on the integration of decellularized nerve grafts and tissue repair in rat models of peripheral nerve defects. Rat and mouse models are often the first choice for nerve regeneration studies. For this study we focused on the rat model because there is a marked predominance of rat use in nerve regeneration studies. Also, because rat nerves are larger and more resilient than the corresponding mouse nerves, standardized functional tests can be performed and the anatomy of rat nerves is better known (Greene, 1935; Tos et al., 2008). A total of 185 studies were retrieved, of which 149 full-text articles were excluded: 25 review articles; 30 articles because they concerned decellularization of tissue or matrices other than nerves; 12 studies on engineered nerve tissue with different cell sources unrelated to any decellularization protocol; 12 studies on the repair of spinal cord injuries but not peripheral nerve lesions; 23 studies on the clinical use of commercial products for which decellularization techniques are not described; 9 studies described only *in vitro* approaches and investigations; 7 were unrelated to nerve regeneration and/or integration; 8 involved animal species other than rats; 26 lacked specific control groups, such as the absence of autograft or pure decellularized nerve graft implantation, making it impossible to verify the efficacy of the decellularization protocols compared to the selected studies. Ultimately, a total of 33 studies performed in rat models met our inclusion criteria for this review of the literature (Figure 1 and Table 1).

**Table 1:** *In vivo* studies of nerve tissue decellularization and graft implantation.

Decellularization protocol	Implantation time (week)	Graft length (mm)	Investigations	Results	Reference
SP; HP; FTP	52	15	Sciatic functional index	AG = HP HP > FTP and SP	Nagao et al., 2011
SP	8	10	Conduction velocity, latency period, wave amplitude, muscle wet weight ratio, myelinated fiber density, axon diameter, myelin sheath thickness	AG > SP	Jia et al., 2012
SP	2,12	20	Muscle wet weight and tension ratio, myelinated fiber number, myelin sheath thickness, Von Frey hair sensitivity test	AG > SP	Wang et al., 2012
SP	1,2,4,8,12	15	Sciatic functional index, conduction velocity, myelinated fiber density, myelinated fiber number, myelin sheath thickness, immunostaining	AG > SP	Zhou et al., 2014
SP	8	10	Sciatic functional index, conduction velocity, latency period, wave amplitude, muscle wet weight ratio, myelinated fiber number, myelin sheath thickness	AG > SP	Zhang et al., 2014
SP	12	15	Sciatic functional index, conduction velocity, muscle wet weight and tension ratio, myelinated fiber density, axon diameter, myelin sheath thickness, immunostaining, gene expression (nerve growth factor, glial-derived neurotrophic factor, growth-associated protein 43, neurofilament heavy)	AG > SP, AG = SP in the first 6 weeks for sciatic functional index. Expression of all genes: AG > SP	Zheng et al., 2014
SP	20	6	Latency period, wave amplitude, myelinated fiber density, axon diameter, myelin sheath thickness	SP = AG	Zhu and Weihua, 2014
SP	1,2,4	15	Myelinated fiber density, axon diameter, myelin sheath thickness Immunostaining Gene expression (angiogenesis-related genes)	AG > SP Expression of angiogenesis-related genes: AG > SP	Zhu et al., 2015
SP	12	10	Conduction velocity Myelinated fiber density, myelinated fiber number, axon diameter, myelin sheath thickness Immunostaining	AG > SP, AG = SP myelinated fiber number Number of Schwann cells: AG > SP	Huang et al., 2015
SP	15	15	Sciatic functional index Immunostaining	AG = SP Presence of myelin in AG but not in SP	Garcia-Pérez et al., 2017
SP	8	10	Latency period, conduction velocity, wave amplitude Muscle wet weight ratio Myelinated fiber density, axon diameter, myelin sheath thickness	AG > SP Presence of laminin and myelin in AG, in SP only laminin	Jia et al., 2017
SP	1,2,3,4	10	Immunostaining	AG > SP	Zhu et al., 2017
HP + NP	6,12,22	14,28	Sciatic functional index Muscle wet weight ratio Myelinated fiber number, axon diameter	14-mm graft: AG = HP + NP, AG > HP + NP for myelinated fiber number at 6 weeks. AG = HP + NP at 12 weeks 28-mm graft: AG > HP + NP at 6 and 22 weeks HP + NP resulted non-immunogenic and maintained laminin structure	Whitlock et al., 2009
HP	10,20	20,40,60	Muscle wet weight and tension ratio Myelinated fiber number, axon diameter Immunostaining Gene expression (senescence markers)	AG > HP Senescence: AG < HP	Saheb-Al-Zamani et al., 2013
HP	6,12	10	Sciatic functional index Conduction velocity Muscle wet weight ratio Axon diameter, myelin sheath thickness	AG > HP	Gao et al., 2014a
HP + NP; AP-W	12	35	Muscle wet weight and tension ratio Myelinated fiber number, myelin sheath thickness	AG > HP + NP and AP-W, HP + NP = AP-W	Vasudevan et al., 2014
HP + NP	12	14	Myelinated fiber number, axon diameter, myelin sheath thickness Retrograde nerve tracking	AG > HP + NP, AG = HP + NP for myelinated fiber number Regenerating nerves: AG = HP + NP, allograft > xenograft	Wood et al., 2014
HP	10	20	Myelinated fiber density, myelinated fiber number, axon diameter	AG > HP	Hoben et al., 2015
HP	8	30	Muscle wet weight ratio Myelinated fiber density, myelinated fiber number	AG > HP	Marquardt et al., 2015
HP	8	20	Muscle wet weight and tension ratio Myelinated fiber number Gene expression (Glial-derived neurotrophic factor)	AG > HP, AG = HP for muscle tension ratio Glial-derived neurotrophic factor expression: AG > HP	Yan et al., 2016
HP	8	10	Myelinated fiber number, axon diameter, myelin sheath thickness Retrograde nerve tracking	AG = HP, AG > SP for myelinated fiber number Regenerating neurons: AG > HP	Tajdaran et al., 2016
HP; HP + NP	12	15	Conduction velocity Muscle wet weight ratio Myelinated fiber density, myelin sheath thickness	AG > HP and HP + NP, HP + NP > HP, HP + NP = HP for myelin sheath thickness	Jiang et al., 2016
HP; AP-T	12	15	Muscle wet weight ratio Myelinated fiber density, myelin sheath thickness	AG > HP and AP-T, AP-T > HP	Kim et al., 2016
HP; AP-TPA Triton-X200 in sulfobetaine 10 + sulfobetaine 16 + 4% sodium deoxycholate + 0.1% peracetic acid	1,8	15	Muscle wet weight ratio Myelinated fiber number, axon diameter, myelin sheath thickness Thermosensitivity Immunological response	AG = AP-TPA, AG and AP-TPA > HP, AP-TPA = HP for myelinated fiber number *In vivo* host immune response: AP-TPA < HP	Cai et al., 2017
NP	8,12	20	Sciatic functional index Muscle wet weight and tension ratio Myelinated fiber number, axon diameter, myelin sheath thickness ELISA (collagen I and III)	AG = NP Collagen III expression: AG = NP Collagen I: AG < NP	Wang H. et al., 2016
FTP (5 cycles)	10	10	Myelinated fiber number Unmyelinated/myelinated axons ratio, area of individual myelinated axon, number of axons in unmyelinated bundle	AG = FTP	Godinho et al., 2013
AP-T	3 days,12	15	Sciatic functional index Conduction velocity, latency period, wave amplitude Myelinated fiber number, axon diameter, myelin sheath thickness Immunostaining	AG > AP-T	Liu et al., 2011
AP-T	12	15	Conduction velocity Muscle wet weight ratio Immunostaining	AG > AP-T Presence of acetyl cholinesterase-positive nerve fibers in motor endplates in AG and AP-T	Zhang and Lv, 2013
AP-TS	4,24	10,15	Latency period, wave amplitude Myelinated fiber density, myelin sheath thickness, axon diameter Immunostaining Von Frey’s hair sensitivity test, toe spread factor	AG = AP-TS Basal lamina preserved in AG and AP-TS Limited presence of macrophage in both AG and AP-TS	Wakimura et al., 2015
AP-TS	4,24	10,15	Latency period, wave amplitude Myelinated fiber density, axon diameter, myelin sheath thickness Immunostaining Von Frey’s hair sensitivity test, toe spread factor	AG = AP-TS Basal lamina preserved in AG and AP-TS Limited presence of macrophage in both AG and AP-TS	Wang W. et al., 2016
AP-TS	25	15	Latency period, wave amplitude Myelinated fiber density, axon diameter Von Frey’s hair sensitivity test, toe spread factor	AG = AP-TS Basal lamina preserved in AG and AP-TS	Kusaba et al., 2016
AP-T	11	10	Muscle wet weight and tension ratio Axon diameter, myelin sheath thickness	AG > AP-T, AG < AP-T for axon diameter	Wang et al., 2017b
AP-T	2,12	15	Sciatic functional index Conduction velocity, latency period Muscle wet weight ratio Retrograde nerve tracking	AG > AP-T, AG = AP-T for sciatic functional index at 2 weeks Cellularity and regenerating fibers: AG > AP-T	Xiang et al., 2017

*AG, Autograft or isograft; HP, Hudson Protocol; NP, Neubauer Protocol; SP, Sondell Protocol; FTP, Freeze/Thaw Protocol; AP-T, Authors’ Protocol using Triton X; AP-TS, Authors’ Protocol using Triton X + SDS; AP-TPA, Authors’ Protocol using Triton X + Peracetic Acid; AP-W, Authors’ Protocol using Wallerian degeneration.*

## Results and Discussion

### Decellularization Protocols

Several studies described the attempt to repair severe nerve damage by means of biologically derived scaffolds, such as decellularized nerve grafts. There are three main decellularization protocols for the creation of a functional graft: the one described by Sondell et al. (1998) and Hudson et al. (2004), and the combined Hudson protocol added with chondroitinase ABC by Krekoski et al. (2001). SP was used in 33% of the studies (Jia et al., 2012, 2017; Wang et al., 2012; Zhang et al., 2014; Zheng et al., 2014; Zhou et al., 2014; Zhu and Weihua, 2014; Huang et al., 2015; Garcia-Pérez et al., 2017; Zhu et al., 2015, 2017); HP was used in 24% (Saheb-Al-Zamani et al., 2013; Gao et al., 2014a; Hoben et al., 2015; Marquardt et al., 2015; Kim et al., 2016; Tajdaran et al., 2016; Yan et al., 2016; Cai et al., 2017), and a combined HP was used in 12% (Whitlock et al., 2009; Vasudevan et al., 2014; Wood et al., 2014; Jiang et al., 2016). Other studies (6%) used a FTP with or without the addition of chondroitinase ABC as introduced by Krekoski et al. (2001; Godinho et al., 2013; Wang H. et al., 2016). Moreover, others still (21%) developed an in-house decellularization protocol and tested its efficacy against autografts (Liu et al., 2011; Zhang and Lv, 2013; Wakimura et al., 2015; Kusaba et al., 2016; Wang W. et al., 2016; Wang et al., 2017b; Xiang et al., 2017). One study (3%) compared the efficacy of Hudson’s, Sondell’s, and the FTP in the same experimental setup (Nagao et al., 2011).

The most widely used protocol was developed by Sondell et al. (1998) (SP), who demonstrated *in vitro* good removal of the myelin sheath and cells, along with satisfying nerve regeneration in the absence of inflammatory response *in vivo*. This method is based on two sequential steps with a 3% Triton X-100 solution followed by a 4% sodium deoxycholate solution, in which the combination of a non-ionic surfactant with an anionic detergent is considered efficient to chemically lyse cells.

Several years later, Hudson et al. (2004) proposed a milder chemical treatment to better preserve the structure of the extracellular matrix (ECM). HP requires several steps repeated twice. In particular, an amphoteric detergent consisting of 125 mM sulfobetaine-10 (SB-10) solution is followed by an anionic detergent solution of 0.14% Triton X-200 and 0.6 mM sulfobetaine-16 (SB-16). The use of detergents with a demonstrated mild toxicity at low concentrations allowed Hudson and co-workers to efficiently disrupt the cell membranes while maintaining the ECM intact.

In order to eliminate chondroitin sulfate proteoglycans, which are known to inhibit axonal growth and the growth-promoting cues derived from the ECM components, Krekoski et al. (2001) introduced a step of proteoglycan degradation by means of the chondroitinase ABC enzyme, thus developing the so-called NP. By adopting this improvement, many authors were able to optimize their protocols by adding this degradation step at the end of the decellularization process, especially after HP and the FTP. The original NP started the process with thermal decellularization, which is one of the most common methods to produce biological acellular grafts. In this process, tissues usually undergo three to five repeated freeze–thaw cycles.

In the various in-house protocols (Author’s protocol, AP), three chemicals are employed to obtain nerve decellularization: Triton-X100 or 200 (Liu et al., 2011; Zhang and Lv, 2013; Kim et al., 2016; Wang et al., 2017b; Xiang et al., 2017) (AP-T; 50% of all AP), its combination with SDS (Wakimura et al., 2015; Kusaba et al., 2016; Wang H. et al., 2016) (AP-TS, 30% of AP) or with peracetic acid (Cai et al., 2017) (AP-TPA, 10% of AP). Moreover, Vasudevan et al. (2014) cultured explanted nerves *in vitro* with normal culture media to initiate the Wallerian degeneration and then in PBS to decellularize them and abruptly remove nutrient supply (AP-W, 10% of AP). SDS is an anionic surfactant with amphiphilic properties that lyses cells and denatures proteins. Peracetic acid is a potent oxidizing agent used to sterilize collagen tissues (Kemp, 1994; Gilbert et al., 2006). In decellularization, it acts by enhancing tissue permeability, allowing detergent penetration and subsequent cell colonization (Bottagisio et al., 2016).

The first parameter in the choice of a decellularization protocol is its duration. Duration is important for the time needed to obtain the results, for the structural maintenance of the biological scaffolds, and for the cost of the procedure. In fact, the reduction of the entire process diminishes or eventually avoids the need for sterilization techniques, such as gamma irradiation, which has a controversial effect on decellularized matrix integrity (Boriani et al., 2017). Another issue is the use of numerous chemical detergents with reduced incubation times during the decellularization protocol. Indeed, the combination of different chemicals augments the deprivation of ECM components (Alhamdani et al., 2010) but also permits use of lower doses of detergents and shortens the incubation time, thus facilitating the complete removal of detergents, which results in a more suitable graft for cell repopulation (Rieder et al., 2004; Serghei et al., 2010; Crapo et al., 2011) and makes the procedure less time-consuming. In our analysis of the original version of the protocols considered here, the FTP was found to be the quickest and least laborious: it consists of three to five brief freeze and thaw cycles and takes a few minutes to complete without the need for chemical detergents and devoid of chemical remains with toxic side effects (Serghei et al., 2010; Crapo et al., 2011). All the other protocols take considerably longer: the HP needs almost 69.5 h and the SP 86 h to complete. Besides its shorter duration, the HP requires a lower percentage of chemical detergents, making their elimination more easy. The NP entails the chondroitinase ABC incubation step, which lasts 16 h, so the total duration of decellularization before this step is increased by 16 h. In the group of AP, the AP-TPA was the quickest protocol, taking 84.5 h, similar to the SP. The AP-T protocols varied in the amount of time required for decellularization: from 70 h in the protocol developed by Xiang et al. (2017) to 200 h in the protocol by Wang et al. (2017b), with an average duration of 128 h. The longest protocol was based on Wallerian degeneration developed by Vasudevan et al. (2014) which required 2 weeks of culture in complete culture medium and then another week of incubation in PBS in order to deprive cells of nutrients.

### Evaluation of Parameters to Measure and Compare Nerve Decellularization Protocols After *in vivo* Implantation

The efficacy of nerve regeneration with decellularized nerve grafts was evaluated using quantitative analyses and standardized parameters to compare study outcomes after *in vivo* transplantation in rat models. We examined the SFI, the von Frey hair sensitivity test, and the toe spread factor which, altogether, indicate functional recovery after nerve grafting. The SFI comprises values between -100 (complete loss of functionality) to 0 (normal functionality). The von Frey hair sensitivity test entails stimulation of the animal’s paw by means of increasingly stiff nylon monofilaments; paw withdrawal and flinch are considered positive responses and the test results are presented as a correlation between stimulus intensity and type of response (Chaplan et al., 1994). The toe spread factor is calculated on the basis of the distance from the first to the fifth toe (Geuna et al., 2009).

Among the various electrophysiological tests that evaluate *ex vivo* recovery of the graft‘s electrical functionality after implantation, conduction velocity (CV, m/s) through the graft and compound muscle action potential (CMAP, mV), and two features derived from CMAP, i.e., latency period (μsec) and wave amplitude (mV), were analyzed. CMAP records the summation of all muscle fiber action potentials derived from the activation of a group of motor neurons within a nerve bundle by means of a brief electrical stimulation. The latency period is the time between the application of an electrical stimulus to a nerve and muscle contraction. The wave amplitude represents the maximum value measured from baseline in the CMAP-resulting curve.

To assess muscle atrophy and functional recovery of the muscles most commonly analyzed (gastrocnemius, triceps surae, extensor digitorum, and tibialis anterior), muscle wet weight and maximum contraction tension were considered. Both parameters were analyzed by the studies *ex vivo* and mainly expressed as the ratio between the operated and the uninjured side. Moreover, since functional recovery is based on restoration of physiological neuronal morphology (Geuna et al., 2009), histomorphometric parameters were examined, including myelinated axon number, density (number of fibers/mm^2^), total fiber number, axon diameter (μm), and myelin sheath thickness (μm).

### Morphological and Functional Comparison of Autografts and Sham Control of Nerve Gap Reconstruction in Rat Models

For a quantitative assessment of the functional and morphological recovery of the nerve graft, all manuscripts published in the literature and reporting numerical values for nerve recovery in terms of functionality and morphology over time were analyzed. Specifically, three different time points were considered: 2–5, 6–9, and 10–15 weeks after implantation. Comparable results from week 2 after graft implantation – the time in which nerve regeneration begins - until week 15 after surgery were evaluated. After surgical grafting, 3–6 weeks are necessary for Schwann cells and macrophages to arrive at the injury site and clear it of cellular and myelin debris (Geuna et al., 2009). The Schwann cells then start to proliferate, organizing themselves in columns to permit the association of the regenerating axons. The choice of 15 weeks as the last time point was based on the study by Waitayawinyu et al. (2007), who reported that analyzing the outcomes of nerve recovery beyond 15 weeks may lead to misleading interpretations because differences between the experimental groups might be lost, also given the high regenerative capacity of the rodent model.

Most studies (Liu et al., 2011; Wang et al., 2012; Zhang and Lv, 2013; Gao et al., 2014a; Vasudevan et al., 2014; Wood et al., 2014; Zheng et al., 2014; Zhou et al., 2014; Huang et al., 2015; Jiang et al., 2016; Kim et al., 2016; Wang H. et al., 2016; Xiang et al., 2017) (39% of the studies included) set week 12 as the last endpoint and a few went beyond the 15th week. For instance, Zhou et al. (2014) and Saheb-Al-Zamani et al. (2013) evaluated the regenerative process until week 20, Whitlock et al. (2009) until week 22, Wakimura et al. (2015), Kusaba et al. (2016), and Wang W. et al. (2016) until week 24, and Nagao et al. (2011) until week 52.

As autografting represents the gold standard procedure for peripheral nerve reconstruction, we first analyzed its outcomes and then compared them with those obtained from sham procedures on healthy nerves. Only five studies included a sham control (Nagao et al., 2011; Tajdaran et al., 2016; Wang H. et al., 2016; Wang et al., 2017b; Garcia-Pérez et al., 2017), in which a slight decrease in nerve functionality after sham surgery (sham control) was found compared to that of the native nerves. This small number of studies could constitute a limitation of the present literature review and preclude comparison of the parameters analyzed. Taking into account that evaluation based on the SFI could vary widely depending on artifacts and operator ability (Varejao et al., 2001; Nichols et al., 2005; Monte-Raso et al., 2008; Fricker et al., 2016), the mean SFI of the sham controls was -8.16 at week 15 (Nagao et al., 2011; Wang H. et al., 2016; Wang et al., 2017a; Garcia-Pérez et al., 2017). Only one study reported a SFI of -2, which approached the normal value at 52 weeks after surgery (Nagao et al., 2011). The SFI of autografts was expected to be lower than the sham surgery. The average SFI values were, in fact, significantly lower for the autografts than the sham controls at all time points. These data indicate that also sham surgery affects nerve functionality, albeit to a lesser extent. Differently, although autografting compromised nerve functionality initially, there was a predictable improvement in the SFI over time, indicating that nerve regeneration and recovery processes had begun.

**FIGURE 2:**
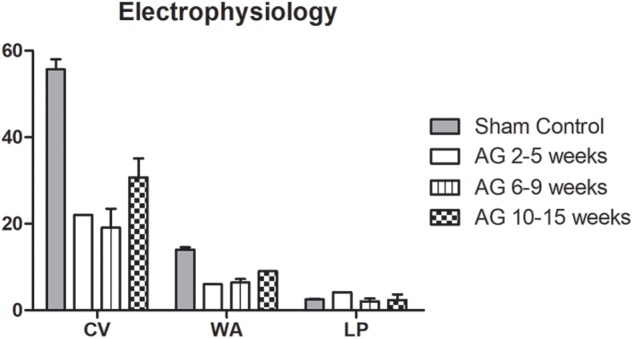
Average values of electrophysiological parameters indicating the electrical functionality of nerves. The Conduction Velocity (CV; m/s), Wave Amplitude (WA; mV) and Latency Period (LP; ms) are compared between sham control and autografts (AG) at different time points. Data are reported as mean ± SD.

The electrophysiological parameters (CV, WA, and LP) reported in Figure 2 were compared among studies, obtaining data for the sham controls and AG 2–5 weeks from one study (Wang H. et al., 2016), for AG 6–9 weeks from four studies (Jia et al., 2012; Zhang et al., 2014; Wang H. et al., 2016; Jia et al., 2017), and for AG 10–15 weeks from six studies (Liu et al., 2011; Zhang and Lv, 2013; Zheng et al., 2014; Huang et al., 2015; Jiang et al., 2015; Wang H. et al., 2016). The low amount of recorded data did not permit to statistically analyze the differences in the results. However, the trend of the electrophysiological parameters (Figure 2) showed higher CV and wave amplitude values for the sham control, while the latency period in the autografts was longer during the first 5 weeks after surgery and almost the same for the two groups at the later time points. At 6–9 weeks, this parameter was even lower for the autografts, probably because only one study reported data for the sham control (Wang H. et al., 2016). The three parameters showed improvement over time, with an increase in CV and wave amplitude at the last time point, reaching half that of the sham control values. Otherwise, the latency period shortened over time, similar to the sham control value.

Muscle functionality, reported as the muscle wet weight and tension ratio, was greater in the sham control compared to autografts at all time points. The average muscle wet weight ratio in the sham control was 82% of that of the native limb (Wang H. et al., 2016), while the ratio obtained with the autografts was increased over time, from a mean of 46.3–51.6% from weeks 6–9 to 10–15, though none of the studies analyzed this parameter during the first 5 weeks (Whitlock et al., 2009; Liu et al., 2011; Jia et al., 2012, 2017; Wang et al., 2012, 2017a; Zhang and Lv, 2013; Vasudevan et al., 2014; Zheng et al., 2014; Marquardt et al., 2015; Jiang et al., 2016; Kim et al., 2016; Wang H. et al., 2016; Yan et al., 2016; Cai et al., 2017). The average muscle tension was 75% for the sham control and 54.7% for the autografts at the last time point; no analyses were performed at earlier time points. These results demonstrated that the autografts supported and strengthened reinnervation along the graft over time, resulting in a decrease in muscle atrophy and reinforcement of the tension that the muscles were able to elicit.

**FIGURE 3:**
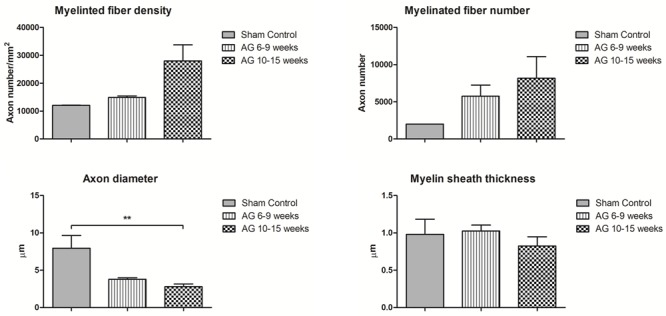
Quantitative analysis of the neuronal morphology based on different parameters emerging from the histomorphometric evaluations. The myelinated fiber density (number of axons per mm^2^), myelinated fiber number, axon diameter (μm) and myelin sheath thickness (μm) are compared between sham control and autografts (AG) at different time points. One way ANOVA for nonparametric data and Dunns’ *post hoc* correction was performed. Data are reported as mean ± SD, ^∗∗^*p* < 0.01.

In the case of histomorphometric analyses, several authors reported evaluable data to be analyzed through non-parametric statistical tests, as reported in Figure 3. Indeed, for the sham controls, five authors reported quantitative parameters (Jia et al., 2012, 2017; Zhang et al., 2014; Wang H. et al., 2016; Wang et al., 2017b). For AG at 6–9 weeks, seven records indicated values for myelin and axon morphology (Whitlock et al., 2009; Jia et al., 2012; Zhang et al., 2014; Marquardt et al., 2015; Tajdaran et al., 2016; Cai et al., 2017; Jia et al., 2017), as well as, fourteen records detailed values for AG at 10–15 weeks (Whitlock et al., 2009; Liu et al., 2011; Wang et al., 2012, 2017b; Godinho et al., 2013; Vasudevan et al., 2014; Wood et al., 2014; Zheng et al., 2014; Zhou et al., 2014; Hoben et al., 2015; Huang et al., 2015; Jiang et al., 2015; Kim et al., 2016; Wang H. et al., 2016).

As expected, myelinated fiber density and number were lower in the sham control than with the autograft at 6–9 and 10–15 weeks (Figure 3). The axon diameter in the sham controls showed a normal opposite trend, with a significant difference compared to the autografts at 10–15 weeks (*p* < 0.01). Unexpectedly, myelin sheath thickness was comparable between the sham controls and autografts and over time was decreased for the autografts. These data are far higher than normal estimated for a regenerating nerve, in which myelin sheath thickness is expected to be lower than in sham controls and directly proportional to the regenerating axon diameter, especially during the first weeks of regeneration.

There was a fluctuating trend for myelinated fiber density in the autografts owing to the study by Zhou et al. (2014) who reported a value of 53876 at weeks 2–5 (data not shown). Moreover, the myelinated fiber density in the autografts continued to increase, reaching 29000 myelinated fibers/mm^2^ at 24 weeks, as reported by Wakimura et al. (2015) and Wang W. et al. (2016). These authors also reported an average myelin sheath thickness of 1 μm at 24 weeks after surgery (Wakimura et al., 2015; Wang W. et al., 2016). In addition, the axon diameter increased, reaching 5.6 μm (Wakimura et al., 2015; Kusaba et al., 2016; Wang W. et al., 2016). The data reported in Figure 3 do not include the results reported by Godinho et al. (2013) because they seem to differ greatly (23000 myelinated fiber number) from the physiological parameters of a healthy nerve.

Interestingly, Godinho et al. (2013) evaluated three more parameters that could be useful for gaining a broader perspective on nerve function recovery at 8 weeks after surgery. The first parameter was the unmyelinated and myelinated axon ratio, which was 6 in the sham control and 3 in the autograft. The second was the average number of axons in unmyelinated bundles, which was 3.5 in both groups. Moreover, in the same study, the sectional area of the myelinated axons was greater in the sham control than in the autograft group (9.5 vs. 3.6 μm^2^).

Overall, most of the studies reported consistent and comparable results for SFI, muscle wet weight ratio, and wave amplitude. Only a few reported values that substantially differed from the mean. In the autograft group, Wang et al. (2017b) reported a SFI markedly lower than average at all three time points, with a value of -48 at weeks 2–5, -45 at weeks 6–9, and -40 at weeks 10–15. Zhang et al. (2014) reported a much higher SFI and muscle wet weight ratio, with an increase from –32.3 after 2 weeks to –18.7 after 8 weeks and a muscle weight ratio of 89.5% at 8 weeks after surgery. Also, Xiang et al. (2017) reported a SFI value higher (-17) compared to the autograft average at 12 weeks. Liu et al. (2011) found a wave amplitude of 29.5 mV at 12 weeks after autografting, which was twice the mean value obtained for the sham control.

As expected, the overall values for the autografts were lower than the sham controls. In the majority of the parameters, there was a positive trend for the autografts over time, indicating a progressive recovery in nerve functionality and morphology. This recovery is due to the autografts providing viable Schwann cells and neurotrophic factors that support nerve axon regeneration (Flores et al., 2000; Siemionow and Brzezicki, 2009). Autografts are, in fact, effective and employed in the treatment of severe nerve injury in clinical practice (Rinker and Vyas, 2014). Based on this premise, the outcomes with autografts were compared with the results obtained with the different decellularization protocols and were considered as benchmark values for evaluating the recovery of graft functionality and morphology.

### Morphological and Functional Outcomes of Nerve Grafts Obtained With Decellularization Protocols

In order to evaluate the functional and morphological efficacy of decellularized nerve grafts at the three time points (2–5, 6–9, and 10–15 weeks after implantation), these parameters were analyzed and compared with respect to the decellularization protocols and to autografts or isografts used as the gold standard.

Also, the implanted graft length was considered. The success of regeneration has been shown to be proportional to the extent of the interstump zone and of the advancing sprouting cell protrusions. Nerve regeneration takes place through the extension of Schwann cell protrusions rather than axonal growth; Schwann cells guide and support the regenerating axons and regulate the rate of the recovery process (Son and Thompson, 1995). Lundborg et al. (1982) experimentally fixed at 10 mm the length beyond which the nerve injury is considered a critical defect in the rat model. This limit was corroborated by Saheb-Al-Zamani et al. (2013) who found it difficult to regenerate with the use of decellularized grafts longer than 20 mm and reported no regeneration in grafts in 60 mm long. Therefore, graft length is a crucial feature, as are its functional and morphological recovery properties, for developing a suitable alternative to autografts. Moreover, as stated by Hare et al. (1992) and demonstrated by Whitlock et al. (2009), the use of a very long nerve graft (28 mm) could produce misleading results in the evaluation of nerve functionality due to the poor reinnervation process along the distal edges of the grafts.

In the studies that used the classic decellularization protocols (SP, HP, NP, and FTP), the majority (75.8%) used grafts ranging in length from 10 to 15 mm (Whitlock et al., 2009; Liu et al., 2011; Nagao et al., 2011; Jia et al., 2012, 2017; Godinho et al., 2013; Zhang and Lv, 2013; Gao et al., 2014a; Wood et al., 2014; Zhang et al., 2014; Zheng et al., 2014; Zhou et al., 2014; Huang et al., 2015; Wakimura et al., 2015; Zhu et al., 2015; Jiang et al., 2016; Kim et al., 2016; Kusaba et al., 2016; Tajdaran et al., 2016; Wang W. et al., 2016; Wang et al., 2017b; Cai et al., 2017; Garcia-Pérez et al., 2017; Xiang et al., 2017). Among the others, only Zhu and Weihua (2014) used a shorter graft (6 mm), while others still used longer grafts: a 20-mm graft in five studies (Wang et al., 2012; Saheb-Al-Zamani et al., 2013; Hoben et al., 2015; Yan et al., 2016; Wang H. et al., 2016); a 30-mm graft in two (Whitlock et al., 2009; Marquardt et al., 2015); a 35–40 mm graft in two (Saheb-Al-Zamani et al., 2013; Vasudevan et al., 2014), and a 60- mm graft in one study (Saheb-Al-Zamani et al., 2013). The mean graft length used with the SP was 12.6 mm, 16.7 mm with the HP, 21 mm with the NP, and 12.5 mm with the FTP. Regarding the various in-house protocols, the average graft length was 14.3 mm (range, from 10 to 15 mm), except for the 35-mm-long graft developed by Vasudevan et al. (2014). The average length of the AP-T grafts was 14 mm and that of the AP-TS and AP-TPA grafts was 15 mm. In general, the average length of the autografts and isografts was 14.8 mm.

#### Sciatic Functional Index

Like the autografts, all the decellularized grafts, except for those produced using the NP, showed an improvement in the SFI (Table 2). In particular, the results of the grafts produced using the SP seemed to be closer to those obtained with the autografts. It has to be taken into account, however, that the average SP graft length was 4 mm shorter than that of the autografts or the HP grafts and 9 mm shorter than the NP grafts. This difference could influence the SFI, although no studies examined whether length difference had an impact on the SFI. Zhang et al. (2014) reported a value of -33.39 for SP grafts at 8 weeks after surgery, a value much higher than the SP or the autograft average. Only two studies calculated the SFI of the NP grafts (Whitlock et al., 2009; Wang H. et al., 2016a). Wang H. et al. (2016) reported higher positive values than Whitlock et al. (2009) which could explain the opposite trend observed in this protocol. Only Nagao et al. (2011) calculated the SFI in HP and FTP grafts, while only Liu et al. (2011) evaluated it in AP-T grafts. This lack of relevant data precluded analysis of this parameter. Therefore, our analysis focused on the study by Nagao et al. (2011) who evaluated and compared the SFI for the SP, HP, and FTP grafts in the same experimental procedure. They found that the grafts reached a plateau in the recovery of SFI at 12 weeks, and that from week 12 until week 52 the SFI for the HP grafts was statistically superior compared to the SP and FTP grafts. In brief, a more rapid recovery during the first weeks after surgery was noted for the HP grafts, which then maintained a higher SFI over time.

**Table 2:** Average values of the Sciatic Functional Index evaluated from 2 to 15 weeks after surgery.

	Sciatic Functional Index								
		Well-established protocols				Authors’ (in-house) protocols			
Time point	AG	SP	HP	NP	FTP	AP-T	AP-TS	AP-TPA	AP-W
2–5 weeks	–83.9 (Whitlock et al., 2009; Nagao et al., 2011; Zheng et al., 2014; Zhou et al., 2014; Wang H. et al., 2016; Garcia-Pérez et al., 2017)	–88.2 (Whitlock et al., 2009; Nagao et al., 2011; Zheng et al., 2014; Zhou et al., 2014; Wang H. et al., 2016; Garcia-Pérez et al., 2017)	–96.5 (Nagao et al., 2011)	–71.5 (Whitlock et al., 2009; Wang H. et al., 2016)	–97.5 (Nagao et al., 2011)	–94 (Liu et al., 2011)	n.d.	n.d.	n.d.
6–9 weeks	–74.4 (Whitlock et al., 2009; Nagao et al., 2011; Zheng et al., 2014; Zhou et al., 2014; Wang H. et al., 2016; Garcia-Pérez et al., 2017)	–75.1 (Nagao et al., 2011; Zheng et al., 2014; Zhou et al., 2014; Garcia-Pérez et al., 2017)	–86.5 (Nagao et al., 2011)	–74.7 (Whitlock et al., 2009; Wang H. et al., 2016)	–88.5 (Nagao et al., 2011)	–88.5 (Liu et al., 2011)	n.d.	n.d.	n.d.
10–15 weeks	–64.5 (Whitlock et al., 2009; Liu et al., 2011; Nagao et al., 2011; Zheng et al., 2014; Zhou et al., 2014; Wang H. et al., 2016; Garcia-Pérez et al., 2017)	–66 (Nagao et al., 2011; Zheng et al., 2014; Zhou et al., 2014; Garcia-Pérez et al., 2017)	–75.7 (Nagao et al., 2011)	–77 (Whitlock et al., 2009; Wang H. et al., 2016)	–79.5 (Nagao et al., 2011)	–80.5 (Liu et al., 2011)	n.d.	n.d.	n.d.

*The average SFI values of the autograft (AG) are also reported. n.d., not declared.*

Extensively employed in nerve injury and recovery studies, this parameter is generally considered to be highly accurate and reliable in describing sciatic nerve function (Varejao et al., 2001; Nichols et al., 2005). Nonetheless, SFI has certain disadvantages: frequent footprint artifacts and distortions produced by smearing of the ink when applied to the rats’ paws (Fricker et al., 2016); the footprint key points have to be adequately recognized and analyzed, which are operator dependent (Monte-Raso et al., 2008). For these reasons, together with the lack of data, this parameter was insufficient to describe functional recovery, but it was used to complete the evaluation of functional restoration in association with other parameters.

#### Electrophysiology

In our analysis of the electrophysiological features of decellularized nerve grafts, sufficient data for a comparison were available only for CV at 10–15 weeks (Table 3). Electrophysiological measurement allows for a meaningful evaluation of the effective re-innervation of the graft over time. For instance, latency period and CV are correlated with the myelination rate and the size of the regenerating axons (Geuna et al., 2009). The CV can be subject to high variability during recording, however (Geuna et al., 2009). Nonetheless, it is a reliable factor for the evaluation of nerve regeneration (Booth and Gollnick, 1983). The CV in nerve grafts obtained with NP, HP, and AP was nearly half that of the SP grafts, which showed CV values slightly below those of the autografts. CV in the HP and NP grafts was measured only in one study (Jiang et al., 2016). The wave amplitude is correlated with the number and size of effectively regenerated axons, in which electrical impulses can be propagated. Also, CV is influenced by the graft distance (Geuna et al., 2009). The best results were obtained with the AP-T grafts, with higher values than those of the autografts. The amplitude of the SP grafts was half that measured for the autografts, although evaluated at the previous time point (data not shown). Given the longer average length of the AP-T grafts, this outcome was remarkable, notwithstanding its being calculated only by Liu et al. (2011). In contrast, the latency period was shorter in the SP than the AP-T grafts, indicating greater fiber maturation in the SP than in the AP-T grafts (Wang W. et al., 2016) (data not shown).

**Table 3:** Average conduction velocity recorded in decellularized grafts and in autografts (AG).

	Conduction velocity (m/s)								
		Well-established protocols				Authors’ (in-house) protocols			
Time point	AG	SP	HP	NP	FTP	AP-T	AP-TS	AP-TPA	AP-W
10–15 weeks	30.7 (Liu et al., 2011; Zhang and Lv, 2013; Zheng et al., 2014; Huang et al., 2015; Jiang et al., 2016; Wang H. et al., 2016)	27 (Zheng et al., 2014; Huang et al., 2015)	12.2 (Jiang et al., 2016)	15.5 (Jiang et al., 2016)	n.d.	12.9 (Liu et al., 2011; Zhang and Lv, 2013; Xiang et al., 2017)	n.d.	n.d.	n.d.

*Only the values measured at the last time point are reported. n.d., not declared.*

All the studies on AP-TS grafts evaluated the wave amplitude and latency period at 25 weeks after surgery. The results were expressed in percentage (43.2 and 46.9%, respectively) with respect to the contralateral non-operated limb, precluding their comparison with the other groups (Wakimura et al., 2015; Kusaba et al., 2016; Wang W. et al., 2016).

Due to the variability in the CV recordings and the absence of data for wave amplitude and latency period, the electrophysiological parameters need to be correlated with other functional and morphological data in order to evaluate the whole regeneration process.

#### Muscle Functionality

Evaluation of muscle functionality is the key to assessment of the progress of nerve regeneration. The muscle wet weight ratio is related to muscle atrophy: without physiological innervations, muscles would undergo atrophy and loss of functionality and lose weight. Therefore, an increase in muscle weight and tension reflects the effective reinnervation of a graft able to conduct and deliver electrical stimuli to the target effector muscle (Zhao et al., 2014; Wang et al., 2015). The peripheral nervous system is capable of a certain degree of self-regeneration (Cerri et al., 2014). But when nerve continuity is interrupted, and particularly when the gap defect is large, complete reinnervation will be difficult. For this reason, we considered the muscle functionality parameter as being highly relevant for predicting effective functional recovery. Restoration of muscle function implies that the regenerating nerve fibers have effectively crossed the graft and reached the target muscle. In the present analysis, the best result was obtained with AP-TPA grafts, which showed the best muscle mass recovery similar to that of autografts (Table 4). This represents a remarkable result because it was evaluated at 8 weeks after surgery with a 15-mm-long graft (Cai et al., 2017). Unfortunately, no data on muscle tension were reported for AP-TPA grafts. Among the other protocols, the HP grafts came the closest to the autografts at the last time point, with increasing improvement over time and good recovery from muscle atrophy. The NP grafts started at a value similar to the HP grafts at weeks 6–9 but the rate of improvement fell behind that of the HP grafts over the following weeks, probably due to the higher average length of the grafts. In contrast, no increase was observed for the SP grafts. Moreover, Zhu and Weihua (2014) found some motoneuron endplate activity in the SP grafts also on the operated side, but Zheng et al. (2014) reported that these motor endplates were scarce and characterized by serious atrophy. Vasudevan et al. (2014) calculated the muscle wet weight ratio for AP-W grafts and reported the lowest outcomes of all the decellularization protocols.

**Table 4:** The average muscle wet weight ratio evaluated in autografts (AG) and decellularized grafts.

	Muscle wet weight ratio (% on healthy limb)								
		Well-established protocols				Authors’ (in-house) protocols			
Time point	AG	SP	HP	NP	FTP	AP-T	AP-TS	AP-TPA	AP-W
6–9 weeks	46.4% (Whitlock et al., 2009; Jia et al., 2012, 2017; Zhang et al., 2014; Marquardt et al., 2015; Yan et al., 2016; Cai et al., 2017)	33.1% (Jia et al., 2012, 2017; Zhang et al., 2014)	34.8% (Gao et al., 2014a; Marquardt et al., 2015; Yan et al., 2016; Cai et al., 2017)	33% (Whitlock et al., 2009)	n.d.	n.d.	n.d.	47% (Cai et al., 2017)	n.d.
10–15 weeks	51.6% (Whitlock et al., 2009; Liu et al., 2011; Wang et al., 2012, 2017b; Zhang and Lv, 2013; Vasudevan et al., 2014; Zheng et al., 2014; Jiang et al., 2016; Kim et al., 2016; Wang H. et al., 2016)	31.5% (Wang et al., 2012; Zheng et al., 2014)	44.8% (Gao et al., 2014a; Jiang et al., 2016; Kim et al., 2016)	37.3% (Whitlock et al., 2009; Jiang et al., 2016; Wang H. et al., 2016)	n.d.	39.8% (Liu et al., 2011; Zhang and Lv, 2013; Kim et al., 2016; Wang et al., 2017b)	n.d.	n.d.	25% (Vasudevan et al., 2014)

*Only the values at 6–9 weeks and 10–15 weeks are reported. n.d., not declared.*

The muscle tension ratio was evaluated only for the SP, NP, and AP-T grafts at weeks 10–15 (Table 5). It reflected the same trend of the previous parameter, in which the muscle tension ratio for the AP-T grafts was slightly higher than that of the NP grafts, which was greater than that of the SP grafts, albeit to a lesser extent. This parameter was calculated only by one author for both NP (Wang H. et al., 2016) and AP-T grafts (Wang et al., 2017b).

**Table 5:** Average muscle tension ratio evaluated in autografts and decellularized grafts.

	Muscle tension ratio (% in healthy limb)								
		Well-established protocols				Authors’ (in-house) protocols			
Time point	AG	SP	HP	NP	FTP	AP-T	AP-TS	AP-TPA	AP-W
10–15 weeks	54.7% (Wang et al., 2012, 2017b; Vasudevan et al., 2014; Zheng et al., 2014; Wang H. et al., 2016)	42.8% (Wang et al., 2012; Zheng et al., 2014)	n.d.	45% (Wang H. et al., 2016)	n.d.	48.8% (Wang et al., 2017b)	n.d.	n.d.	n.d.

*The reported values were measured only at the last time point. n.d., not declared.*

Further evidence of recovery of muscle functionality was presented by Zhang and Lv (2013) and Zhu and Weihua (2014) who demonstrated, in SP and AP-T grafts, respectively, the presence of fibers positive to acetylcholinesterase immunostaining in some motoneuron endplates.

#### Morphology

Quantitative histomorphology is an essential method to investigate nerve regeneration, as numerous morphological parameters can be associated with functional outcomes (Geuna et al., 2004; Vleggeert-Lankamp, 2007). Table 6 presents the morphological data, in which only values at 10–15 weeks are included since the results at this time point were richer. AP-TPA is an exception because it had better and promising outcomes than the other decellularization protocols. It has been included in the table even if it was evaluated at 6–9 weeks.

**Table 6:** Histomorphometric parameters at 10–15 weeks.

	Histomorphology								
	Well-established protocols					Authors’ (in-house) protocols			
Parameter	AG	SP	HP	NP	FTP	AP-T	AP-TS	AP-TPA	AP-W
Myelinated fiber density (myelinated fibers/mm^2^)	27950.7 (Vasudevan et al., 2014; Zheng et al., 2014; Zhou et al., 2014; Jiang et al., 2016; Kim et al., 2016; Wang H. et al., 2016)	12500 (Zheng et al., 2014)	12748 (Hoben et al., 2015; Jiang et al., 2016; Kim et al., 2016)	6799 (Wood et al., 2014; Jiang et al., 2016)	27000 (Godinho et al., 2013)	9893 (Kim et al., 2016)	n.d.	n.d.	50000 (Vasudevan et al., 2014)
Myelinated fiber number	12939.6 (Whitlock et al., 2009; Godinho et al., 2013; Wood et al., 2014; Huang et al., 2015; Kim et al., 2016)	1400 (Huang et al., 2015)	n.d.	12332 (Whitlock et al., 2009)	n.d.	5211 (Kim et al., 2016)	n.d.	5600^∗^ (Cai et al., 2017)	n.d.
Axon diameter (μm)	2.8 (Liu et al., 2011; Wood et al., 2014; Zheng et al., 2014; Zhou et al., 2014; Hoben et al., 2015; Huang et al., 2015; Wang H. et al., 2016; Wang et al., 2017b)	2.1 (Zheng et al., 2014; Zhou et al., 2014; Huang et al., 2015)	2.7 (Hoben et al., 2015)	3.8 (Wood et al., 2014; Wang H. et al., 2016)	n.d.	1.5 (Liu et al., 2011; Wang et al., 2017b)	n.d.	4.1^∗^ (Cai et al., 2017)	n.d.
Myelin sheath thickness (μm)	0.8 (Liu et al., 2011; Wang et al., 2012, 2017b; Vasudevan et al., 2014; Wood et al., 2014; Zheng et al., 2014; Zhou et al., 2014; Huang et al., 2015; Jiang et al., 2016; Kim et al., 2016; Wang H. et al., 2016)	0.6 (Wang et al., 2012; Zheng et al., 2014; Zhou et al., 2014; Huang et al., 2015)	0.8 (Jiang et al., 2016)	0.6 (Vasudevan et al., 2014; Wood et al., 2014; Jiang et al., 2016; Wang H. et al., 2016)	n.d.	2.1^§^ (Liu et al., 2011; Kim et al., 2016; Wang et al., 2017b)	n.d.	1^∗^ (Cai et al., 2017)	0.6 (Vasudevan et al., 2014)

*^∗^Outcomes with AP-TPA grafts are included though calculated at the previous time point in order to facilitate comparison of the results, as the grafts produced with this protocol showed good recovery. ^§^Off-the-scale data. n.d., not declared.*

##### Myelinated fiber density

The number of myelinated neurons in a regenerating nerve reflects the rate of functional recovery (Geuna et al., 2009). Regenerating nerves are initially characterized by a shortage of myelin, but, thanks to the action of Schwann cells, the fibers will be surrounded in a myelin layer (Flores et al., 2000). Myelinated fiber density is also related to a nerve’s electrophysiological characteristics and provides a meaningful measure of the recovery process (Russell et al., 1996).

The highest myelinated fiber density, comparable to autograft values, was measured in FTP grafts, and it was evaluated only in one study (Godinho et al., 2013). Comparable results were obtained with SP and HP grafts, in which the fiber density was almost half that of the autografts. Some studies published very variable values for myelinated fiber density. Zhu et al. (2015) and Zhou et al. (2014) reported unexpectedly higher density values for SP grafts with respect to autografts at 2–5 (48231) and 10–15 (40523) weeks, while Zhu and Weihua (2014) reported a lower density of 2090 at week 20. The average density of the NP grafts, calculated only at 10–15 weeks, was the lowest among the decellularized grafts.

Among the grafts produced with an in-house protocol, the AP-T grafts had an intermediate value between the SP/HP and the NP grafts. For the same group (AP-T), Liu et al. (2011) reported only 0.03 myelinated fibers per mm^2^ at week 12, considerably lower than those reported by other studies. The myelinated fiber density of AP-W grafts, reported only by Vasudevan et al. (2014), was out of range since it was almost twice that of the autografts. However, the same study reported a density value out of range also for the NP grafts (75000), which was greater than that of the AP-W grafts. The AP-TS grafts had a higher density (17050) than the HP grafts, though this difference might have resulted from the late time point considered in these studies (Wakimura et al., 2015; Kusaba et al., 2016; Wang W. et al., 2016).

##### Myelinated fiber number

Since some studies evaluated either myelinated fiber density or myelinated fiber number, we examined both these parameters even if their functional significance is identical. Unlike myelinated fiber density, myelinated fiber number was similar in NP grafts and autografts. As seen for other parameters, already at weeks 6–9, the fiber number in the AP-TPA grafts was higher than in those produced with other decellularization protocols and was only slightly lower than the autografts (5743 at week 6–9). The grafts with the lowest number of myelinated fibers were produced with AP-T (Kim et al., 2016) and SP (Huang et al., 2015), though only one study evaluated both types of grafts.

##### Axon diameter

The average axon diameter in a regenerating nerve is an important parameter; it is the determining factor that influences CV and defines the capacity of nerves to transmit electrical stimuli (Hoffman, 1995; Assaf et al., 2008). During the regeneration phase in the first weeks after injury, the axon diameter increases. Evaluation of this parameter during the very early phases may be challenging, however, because it is difficult to discern between small successfully regenerated fibers from small atrophic or dying fibers (Ikeda and Oka, 2012). For this reason, we compared the results measured at the last time point. Axon diameter in AP-TS grafts was the largest (5.4 μm) since it was evaluated at week 25. Once again, AP-TPA had the best outcome among the other protocols, although evaluated only at 8 weeks after surgery. This value was even slightly greater than that for autografts measured at the same time point (3.8 μm), meaning that this graft type probably also had a high CV, which was not experimentally measured, however. The NP grafts had the largest diameter, reflecting the outcome derived from CV recording. HP grafts were evaluated only by one study (Hoben et al., 2015), which found an intermediate value, confirming the graft’s average velocity in the conduction of electrical stimuli. The SP grafts were re-innervated by axons with a diameter among the lowest, higher only than that of the AP-T grafts. In addition, Zhu and Weihua (2014) demonstrated that axon diameter was increased slightly in the SP grafts, reaching 2.5 μm at week 20. A discrepancy was noted in the comparison with the CV recorded for SP grafts: while these grafts had the fastest CV, their axon diameter was among the lowest. This cannot be elucidated by the reported data, since the parameters were evaluated by different studies and were nearly comparable, albeit with low variance. Finally, in line with CV outcomes, the axons in the AP-T grafts had the smallest diameter.

##### Myelin sheath thickness

Myelin sheath thickness has been shown to correlate with the functional recovery of regenerating nerves (Kanaya et al., 1996). During the early period after injury, the axon diameter decreases and then later increases over time (Sanders, 1948). The decrease in axon diameter is associated with a mild increase in myelin sheath thickness, maintaining the size of the entire fiber steady. The axons then start to increase their diameter, causing the surrounding myelin sheath to decrease in thickness (Sanders, 1948). The correlation between these two parameters is useful for interpreting the results. For instance, in the AP-TPA grafts, despite measurement at an earlier time point, myelin sheath thickness and axon diameter were nearly comparable to those of the autografts, meaning that these two types of grafts were probably almost at the same regeneration phase. This comparison was not possible with NP grafts, as myelin sheath thickness was evaluated only at the last time point. Its low value, together with the increased axon diameter, suggested that the grafts were further along in the regeneration process than the HP grafts. In fact, axon diameter was smaller, while myelin sheath thickness remained substantially unchanged, indicating that the HP grafts were probably still in an intermediate phase of regeneration. Kim et al. (2016) reported a myelin sheath thickness of 4 μm in HP grafts at 12 weeks after the graft procedure, which was much higher and out of the range, even when compared with autografts. Differently, a slight increase in axon diameter and a slight decrease in myelin sheath thickness was observed in SP grafts, suggesting that they were probably at the beginning of the second phase of regeneration. The AP-TS grafts were re-innervated by large axons (5.4 μm in diameter) and had a lower myelin sheath thickness (0.8 μm), indicating good progress in the regeneration process, as expected, given the time point at which they were evaluated. Conversely, the myelin sheath was thickest in the AP-T grafts, in which the axon diameter was among the smallest. In this case, the myelin sheath thickness reported for this graft type appeared to be outside the expected physiological range, as mentioned in Section “Morphological and Functional Comparison of Autografts and Sham Control of Nerve Gap Reconstruction in Rat Models.” Unfortunately, some of the studies (Liu et al., 2011; Kim et al., 2016) reported unforeseen and off-the-scale values due to possible methodological sampling errors or, more commonly, insufficient methodological information to obtain significant results, as demonstrated elsewhere (Geuna et al., 2004; Geuna and Herrera-Rincon, 2015).

##### Other histomorphological parameters

The unmyelinated/myelinated axon ratio, the number of axons in the unmyelinated bundle, and the sectional area of single myelinated axons were evaluated by Godinho et al. (2013) in FTP grafts at weeks 10–15. The unmyelinated/myelinated axon ratio was also evaluated in AP-TS grafts (Wang W. et al., 2016). Unmyelinated axons are physiologically present in the peripheral nervous system and can result locally abundant, accounting for about 75% of axons in the cutaneous nerves of mammals (Geuna et al., 2009). They are usually small; given the lack of a myelin sheath, they do not permit saltatory conduction, resulting in a lower CV. Both FTP (with an unmyelinated/myelinated axon ratio of 4) and especially AP-TS grafts (with a ratio of 5.3) seemed to be re-innervated by a higher number of unmyelinated neurons compared with autografts, in which the ratio was 3.5. This implies that the CV was slower in these grafts, though evidence for this parameter was not directly evaluated.

Godinho et al. (2013) observed a difference in axon sectional area between FTP grafts and autografts (2.1 μm^2^ vs. 3.6 μm^2^), supporting the hypothesis that CV in FTP grafts is slower. In addition, the two parameters correlating with functional recovery were evaluated in AP-TS grafts (Wakimura et al., 2015; Kusaba et al., 2016; Wang W. et al., 2016): the von Frey hair sensitivity test and the toe spread factor, both as compared to the healthy limb and expressed as a percentage. The mean von Frey hair sensitivity value was 13.8% and the toe spread factor ratio was 46.3%, consistent with the values for autografts measured in other studies (Wang et al., 2012; Wakimura et al., 2015; Kusaba et al., 2016; Wang W. et al., 2016), indicating comparable functional recovery for AP-TS grafts and autografts at week 20.

#### Vascularization and Inflammation

Nerve grafts obtained with decellularization protocols often proved inadequate for clinical application, and this inadequacy was noted to increase linearly with graft length (He et al., 2012, 2014; Hayashida et al., 2015). A chief reason is that, ischemia, degeneration, and necrosis can occur in the non-vascularized parts of long, decellularized nerve grafts, associated with slow regeneration and risk of graft failure (Hayashida et al., 2015; Rbia and Shin, 2017). In conventional approaches with autografts or isografts, revascularization is simpler and more direct, based on reconnection with the existing vasculature (Best et al., 1999). Differently, the native cells in decellularized grafts, including endothelial cells, are removed, thus impeding adequate and rapid support to the decellularized nerve scaffold. Given the importance of this issue, and in order to better understand the process of vascularization in decellularized grafts, we analyzed two studies. Zhu et al. (2017) evaluated patterns of microangiogenesis in decellularized grafts, while Farber et al. (2016) completed the study by Saheb-Al-Zamani et al. (2013) investigating the difference in vascularization between long auto/isografts and decellularized grafts. Zhu et al. (2017) studied short grafts (10 mm in length) and demonstrated that nutrient supply relies on fluid permeation from the surrounding tissues during the early stages after the graft procedure. Microvessels started to grow at the graft extremities and then penetrated into the decellularized nerve matrix along the long axis. Moreover, they calculated that the microvessels grew more rapidly in the autografts than in the decellularized nerves. After 14 days, newly formed microvessels were anastomosed at the midgraft in the autografts AG, whereas the decellularized grafts needed 21 days for this process.

Farber et al. (2016) demonstrated that long nerve grafts (60 mm) need different times for complete vascularization, depending on the approach used. Auto/isografts take about 5 days before the first signs of vascularization in the midgraft appear, whereas decellularized nerves need about 20 days, as shown in Zhu et al.’s (2017) study. The longer time to obtain adequate vascularization might be the cause of cell damage and senescence, oxidative stress, and other numerous pathological processes. Hence, one of the main reasons why better outcomes are achieved with auto/isografts is this faster and higher microvascularization capability of autografts. Angiogenesis and the formation of new vascularization within the nerve graft are two processes necessary for successful nerve regeneration and must be taken into account in the development of functional nerve grafts.

Another fundamental issue in decellularization is graft biocompatibility, i.e., the risk of eliciting an inflammatory response or immunological rejection of the transplanted nerve matrix. During the decellularization process, a great quantity of cells, myelin sheath, and ECM debris is generated, which is theoretically able to activate inflammatory and macrophage response that could damage the basement membrane and thus delay regeneration (Gao et al., 2014b). Moreover, some of these fragments might result immunogenic and able to activate lymphocyte T cells, promoting their migration within the graft and leading to its destruction. Therefore, it is important to consider the pro-inflammatory and immunologic potential of decellularized nerves used as a graft. In the present review, six studies described immunological response to decellualarized grafts (Jia et al., 2011; Godinho et al., 2013; Fan et al., 2014; Jiang et al., 2015; Wakimura et al., 2015; Poppler et al., 2016; Wang W. et al., 2016; Cai et al., 2017; Kaizawa et al., 2017). All six studies used immunostaining to detect T lymphocyte (CD3, CD4, CD8) and macrophage (CD68) activity. The majority of the studies showed a slight increase in these cells as compared with autografts within and in the immediate proximity of the graft, especially during the first days after grafting, though there was no statistical difference between these values. Only Cai et al. (2017) reported a significant difference between autografts and decellularized nerve grafts. Jiang et al. (2015) quantified the serum concentration of inflammatory cytokines (IL-2, TNF-α, and IFN-γ) after a grafting procedure but found no significant increase in inflammatory cytokines in animals grafted with a decellularized nerve graft and those that received an autograft. These outcomes indicated no apparent graft rejection during the early recovery period but only a restrained inflammatory response. This means that decellularized nerves are not subject to the cytotoxic effect of T lymphocytes and macrophage degradation, making them fairly safe for *in vivo* use.

## Conclusion

With this literature review, we wanted to quantitatively analyze the effectiveness of decellularized nerve grafts as possible substitutes for auto/isografts, which currently represent the current gold standard procedure in the treatment of severe nerve injury. We evaluated functional and morphological parameters that reflect the progressive recovery of nerve function. The parameters were selected because they are the ones most commonly used in the studies and the most representative of the regeneration process. Among these parameters, muscle functionality and histomorphometric analysis (axon diameter and myelin sheath thickness) best predicted effective recovery of nerve function. Restoration of muscle weight and tension entails effective crossing of the nerve gap to reach the target muscle. The histomorphometric evaluations not only highlighted the graft’s morphological features but also its functional and electrophysiological characteristics. Overall, histomorphometric evaluations are subject to methodological sampling errors, which may induce bias in the interpretation of the results. We noted, for example, that myelin sheath thickness was frequently overrated, supporting the hypothesis of common errors in the evaluation of histomorphometric parameters, as pointed out in our previously studies (Geuna et al., 2004; Geuna and Herrera-Rincon, 2015). In contrast, SFI evaluation and electrophysiological measurements are useful in the assessment of nerve regeneration, though they, too, may be subject to error and high variability (Monte-Raso et al., 2008; Geuna et al., 2009; Fricker et al., 2016).

The main limitation of the present study was the lack of data for numerous investigations: not all parameters were evaluated for all decellularization protocols at all time points. Moreover, only one evaluation reported by a single study was available in many cases, making complete and exhaustive comparison between the protocols not always possible.

Overall, although reported in only one study (Cai et al., 2017), the most promising outcomes were obtained with the grafts produced with the AP-TPA protocol. This protocol had the best data for muscle wet weight ratio, myelinated fiber number, axon diameter, and myelin sheath thickness, with all values comparable to those of autografts evaluated at 8 weeks after surgery. Moreover, the effectiveness of this protocol was notable also for the length of the graft (15 mm, longer than the critical size) and the time needed for decellularization (the quickest among the in-house protocols and similar to that of SP). Based on the promising results of AP-TPA, further investigations should be carried out to confirm these outcomes.

Among the well-established protocols, NP, especially when performed after HP, seemed to be the most effective. In fact, it resulted in grafts with sufficient muscle weight and tension ratio, axon diameter, myelin sheath thickness, myelinated fiber number, CV, and SFI (until week 9). Given that the NP grafts were the longest on average (21 mm), the outcomes appeared even more remarkable. This protocol is, in fact, used in the manufacture of Avance^®^ processed nerve grafts (AxoGen, Inc., Alachua, FL, United States), which is a decellularized nerve graft currently employed in clinical practice. The protocol was developed with the idea to improve on the effectiveness of the previously developed, well-established protocols (SP and HP) by removing chondroitin sulfate proteoglycans, which were demonstrated to inhibit axonal growth (Krekoski et al., 2001; Jiang et al., 2016); this improvement was confirmed in our analysis. The HP and the SP showed similar results; however, based on specific parameters, mainly muscle functionality, outcomes with the HP grafts were slightly superior. This observation was confirmed by Nagao et al. (2011) who compared SP, HP, and FTP grafts in the same experimental procedure and reported a significantly better improvement in SFI with use of HP grafts as compared to those obtained with the other two protocols. Moreover, HP takes less time to complete than SP. This can be explained by the fact that SP was the first decellularization protocol to be developed, while HP is a later improvement with better preservation of the ECM structure and an easier detergent removal step using lower doses of chemicals (Hudson et al., 2004; Rieder et al., 2004; Alhamdani et al., 2010; Serghei et al., 2010; Crapo et al., 2011). Evaluation of FTP grafts was scarce, since it was evaluated only by two studies (Nagao et al., 2011; Godinho et al., 2013) that reported the lowest values for SFI, statistically lower than the HP grafts (Nagao et al., 2011). Moreover, in the same experimental design, the myelinated fiber density was lower in the FTP than in the NP grafts (Godinho et al., 2013). Three major advantages of FTP are that it is the quickest decellularization protocol, it requires no chemicals, and the effect of decellularization is uniform on the entire tissue, without depending on diffusion or perfusion (Crapo et al., 2011). Nonetheless, the protocol has many limitations, which explains the worse outcomes obtained. First the technique causes rapid tissue expansion and contraction that can damage basal lamina continuity, making axonal regeneration difficult. Moreover, FTP kills cells in the tissue. Since it does not have a washing step, the debris is not eliminated (Gulati and Cole, 1994; Sondell et al., 1998; Szynkaruk et al., 2013), which might activate inflammatory or immunological response (Gao et al., 2014b).

Concerning the other in-house protocols, the AP-T protocol employed a longer time for decellularization and the outcomes were generally slightly worse than with HP, particularly for morphology. Finally, the AP-W protocol ranked last; its long duration (2 weeks) makes it less attractive and effective than the other protocols.

Table 7 summarizes the advantages and disadvantages of the different techniques used to decellularize nerve grafts for potential clinical use.

**Table 7:** Advantages and disadvantages of the various protocols to decellularize nerve grafts.

Decellularization protocol	Advantages	Disadvantages
Sondell (SP)	• SFI similar to autograft • CV similar to autograft	• Motor endplates seriously atrophied and low recovery of muscle functionality • Very few regenerated myelinated nerve fibers
Hudson (HP)	• Low percentage of chemical detergents • Good muscle wet weight ratio at 10–15 weeks • Axon diameter and myelinated sheath thickness comparable to autografts at 10–15 weeks	• Low CV values 10–15 weeks after repair
Neubauer (NP)	• Satisfying SFI • Satisfying muscle weight and tension ratio • Satisfying quantitative histomorphological parameters (myelinated fiber number, axon diameter, myelin sheath thickness)	• CV values halved of that registered in autografts • Low myelinated fiber density
Freeze-and-thaw (FTP)	• Quickest and less laborious protocol, no chemicals are needed	• Low values of SFI • Low myelinated fiber density • Cell debris are not eliminated • Damaged basal lamina and difficult axonal regeneration
Triton-X (AP-T)	• Remarkable electrophysiological outcome (considering the higher average graft length) • Muscle tension ratio recovery similar to autografts	• Long time to the decellularization process • Low values of SFI recovery
Triton-X + SDS (AP-TS)	• Good progress in nerve regeneration (large axons at 25 weeks) • Basal lamina preserved	• Few data comparable to the other protocols
Triton-X + peracetic acid (AP-TPA)	• Quickest protocol between in-house protocols • Muscle mass recovery (muscle wet weight ratio) at 8 weeks similar to autografts • Quantitative histomorphological parameters (myelinated fiber number, axon diameter, myelin sheath thickness) at 8 weeks comparable with those of the autograft at 10–15 weeks	• Only one paper describing this protocol
Wallerian degeneration (AP-W)	• Myelin thickness of regenerating fibers similar to autografts	• Longest protocol • Muscle wet weight ratio recovery half than autografts

In conclusion, decellularized nerve grafts are a promising, alternative to auto/isografts for the treatment of nerve injury, even in the repair of defects with large gaps. They are immunologically safe and some more than others support the functional and morphological recovery of the injured nerve. Although the use of peracetic acid showed very promising outcomes, it needs to be further analyzed. The use of chondroitinase ABC, introduced with NP, seems to effectively improve the outcomes obtained with SP and HP. The use of triton-X alone or in association with SDS did not seem to improve the outcomes with the HP or the SP grafts. The worst results were obtained with physical techniques (freeze/thaw or Wallerian degeneration protocol); therefore, chemicals and washing steps are needed to obtain an effective decellularized nerve graft. Finally, in order to further improve process performance, a recellularization step could be introduced to produce a graft that is able to substitute the current gold standard of auto/isografts.

## Author Contributions

AL, PT, SG, and SR contributed to the conception and design of the study. AL, DD’A, SO, SG, and SR collected and analyzed the data, interpreted the results, and wrote the article. All authors analyzed the data and interpreted the results, and critically revised the manuscript. All authors approved the publication of the contents and verified that all parts of the work were appropriately investigated and described.

## Conflict of Interest Statement

The authors declare that the research was conducted in the absence of any commercial or financial relationships that could be construed as a potential conflict of interest. The handling Editor and author SR declared their involvement as co-editors in the Research Topic, and confirm the absence of any other collaboration.

## Abbreviations

AG: autograft/isograft
AP: authors’ (in-house) protocol
AP-T: authors’ protocol using triton-X
AP-TPA: authors’ protocol using triton-X and peracetic acid
AP-TS: authors’ protocol using triton-X and SDS
AP-W: authors’ protocol using Wallerian degeneration
CMAP: compound muscle action potential
CV: conduction velocity
FTP: freeze and thaw protocol
HP: Hudson’s protocol
NP: Neubauer’s protocol
PBS: phosphate-buffered saline
SDS: sodium dodecyl sulfate
SFI: Sciatic Functional Index
SP: Sondell’s protocol
